# Palliative Care and Mental Health among Pancreatic Cancer Patients in the United States: An Examination of Service Utilization and Health Outcomes

**DOI:** 10.3390/healthcare12080842

**Published:** 2024-04-16

**Authors:** Divya S. Subramaniam, Zidong Zhang, Zachary Timmer, Elisabeth C. DeMarco, Michael P. Poirier, Leslie J. Hinyard

**Affiliations:** Department of Health and Clinical Outcomes Research, Advanced HEAlth Data (AHEAD) Institute, Saint Louis University School of Medicine, St. Louis, MO 63108, USAzachary.timmer@health.slu.edu (Z.T.); michael.poirier@slu.edu (M.P.P.);

**Keywords:** palliative care, advanced cancer, symptom management, psychosocial support, healthcare utilization

## Abstract

Introduction: Palliative care (PC) utilization remains low among pancreatic cancer patients. This study explores the association of PC with mental health service and pharmacotherapy utilization among pancreatic cancer patients.Methods: Retrospective analysis was conducted on a sample of patients in the United States with newly diagnosed pancreatic cancer using Electronic Health Record data from Optum’s Integrated Claims-Clinical data set. Subsequent diagnoses of anxiety and depression and PC consultation encounters were determined using ICD-9/10 codes. Adjusted associations of mental health treatments with PC and patient characteristics were quantified using multiple logistic regression. Results: Among newly diagnosed pancreatic cancer patients (*n* = 4029), those with PC consultations exhibited a higher prevalence of anxiety (33.9% vs. 22.8%) and depression (36.2% vs. 23.2%). Mental health service use and pharmacotherapy varied, with the highest utilization among patients having both anxiety and depression. Treatment pattern was also influenced by age (aOR 1.832 for age <55 vs. 65–70 years). Notably, PC consultations showed no significant effect on the likelihood of documented treatment. Discussion: Our study emphasizes underutilization of PC and MH treatment for pancreatic cancer patients. These findings imply a crucial need for further investigation into palliative care’s role in addressing mental health concerns among pancreatic cancer patients.

## 1. Introduction

Cancer is a complicated health condition with a wide range of symptoms and treatments that can have tremendous effects on a patient’s mental health and overall well-being [1]. For that reason, the management of cancer and associated symptoms presents a substantial challenge for oncology healthcare providers [1]. In recent years, palliative care has emerged as a promising approach to address these challenges [1,2,3] as it has shown positive outcomes in enhancing the quality of life for various types of cancer patients [2,4,5,6,7,8,9]. Despite the established benefits of palliative care for cancer patients, its adoption and utilization among healthcare providers have been relatively low [5,10], leading to potentially harmful consequences for patients with cancer due to a lack of palliative care-associated health interventions [11]. Numerous factors influence treatment and symptom management, including type of cancer [3,7,12,13,14]; however, the impact of cancer type on anxiety and depression remains a topic of debate in the medical community [10,12,15].

Existing literature has highlighted that the presence of anxiety and depression can exacerbate physical symptoms, such as pain, fatigue, weakness, and functional status [1,13,14,15,16], among patients with advanced cancer, therefore, diminishing their overall quality of life [13]. These mental health issues also affect end-of-life care decisions, potentially leading to more aggressive treatments, such as chemotherapy, intensive care unit admission, and ventilation [3]. Additionally, anxiety and depression can further exacerbate other symptoms, creating a compounding effect [1,4,13,14,15]. The complexity associated with comorbid cancer and anxiety/depression underscores the need for a holistic, interdisciplinary approach to supportive care for cancer patients. Despite the well-documented benefits of palliative care in cancer treatment [2,4,5,7,8,9,10,17], there remains a lack of research on the intersection of palliative care and mental health in the context of supportive cancer care.

Pancreatic cancer is medically complex, often diagnosed at later stages [8,12,15], and documents a higher prevalence of severe anxiety and depression symptoms among its patients [3,4,12,13,14], who were less likely to improve after palliative care consultation [7] compared to patients with other types of cancer. This demonstrates that the type of cancer can profoundly influence both symptom burden and palliative outcome. Patients with pancreatic cancer form a vulnerable cohort that necessitates further investigation into the effects of palliative care and mental health. To address this existing gap in research, our study aims to explore the impact of palliative care consultations on the utilization of mental health services and pharmacotherapy among pancreatic cancer patients diagnosed with anxiety and depression within 12 months of their initial cancer diagnosis.

## 2. Materials and Methods

### 2.1. Data Source and Study Participants

This study utilized Electronic Health Record (EHR) data from Optum’s de-identified Integrated Claims-Clinical data set, including healthcare encounters between 1 January 2010 and 31 December 2018. The Integrated data set is an all-payer data set, i.e., data was captured from records of patients who were commercially, publicly, or uninsured, including Medicare and Medicaid beneficiaries. The study examined adults aged 18 years and older, in the United States, with newly diagnosed pancreatic cancer within the studied period. To identify newly diagnosed pancreatic cancer, all the included patients were required to have at least 6 months of activity in the EHR data prior to the date of first pancreatic cancer diagnosis. Among the patients with newly diagnosed pancreatic cancer, the diagnosis of anxiety, depression, or both was determined, and the number of days from the date of first pancreatic cancer diagnosis to the dates of first diagnosis of these mental health issues. Anxiety and depression diagnoses were identified using ICD-9-CM and ICD-10-CM codes (Appendix A. Table A1). Patients who had anxiety or depression diagnosed prior to their diagnosis of pancreatic cancer were excluded from further analysis.

### 2.2. Ethical Considerations

The Saint Louis University Institutional Review Board deemed this to be Non-Human Subjects Research.

### 2.3. Measures

The primary outcome of interest was utilization of mental health services and pharmacotherapy for anxiety and/or depression. Mental health services were identified by examining the encounters of healthcare services provided by the following specialties: Clinical Neuropsychology, Marriage and Family Therapy, Psychiatric Hospital, Psychiatry, Psychiatry and Neurology, Psychoanalysis, Psychology, and Clinical Social Work. In addition, encounters with mental health services were identified by CPT and HCPCS codes (Appendix A. Table A2). Utilization of pharmacotherapy was determined by examining prescriptions of medications for anxiety and depression within 3 years following the diagnosis of pancreatic cancer (Appendix A. Table A3 and Table A4). The generic and brand names and drug classes were consistent with previous studies and informed by an academic psychiatrist collaborator [18]. The absence of either a mental health services visit or pharmacotherapy was considered no treatment. As treatment for anxiety and depression overlaps, no attempt was made to determine the diagnosis that indicated the treatment (particularly in the case of those with both depression and anxiety diagnoses), but rather whether treatment was documented.

The exposure of interest was a palliative care (PC) consultation, determined by encounters with PC services in the EHR records (ICD-9: V66.7, ICD-10: Z51.5). The V66.7 code has been validated in multiple studies on PC for patients with chronic life-threatening illnesses in the U.S (United States), which find that the code is highly specific (>90%) and moderately sensitive (45–89%) [19]. The Z51.5 code needs further validation but is equivalent to V66.7 [20]. The covariates examined at the time of cancer diagnosis included the patients’ age, gender, race, primary payer, and the Charlson–Deyo Comorbidity Index [21,22].

### 2.4. Statistical Analysis

Chi-Square tests were used to assess the distribution of categorical demographics among groups, as well as the prevalence of mental health service utilization, pharmacotherapy, or any mental health treatment among those with and without palliative care consultation. Continuous demographic variables (i.e., age) by exposure were examined with Student *t*-tests. A multiple regression analysis was conducted to quantify the effects of palliative care consultation, gender, age at time of cancer diagnosis, race, and the Charlson comorbidity index on the use of mental health services and pharmacotherapy. Statistical analyses were performed in SAS 9.4 (SAS Institute, Cary, NC, USA). All tests were two tailed with α = 0.05 to determine statistical significance.

### 2.5. Results

In this study, we identified 4029 adult patients who had newly diagnosed pancreatic cancer out of 5266 patients with any record of pancreatic cancer diagnosis from 2010 to 2018. Within this sample, patients were mostly Caucasian (80.5%), while 9.7% were African American, and 9.8% identified as other. The mean age at the time of cancer diagnosis was 68 years old (Standard Deviation: 12.3 years), while the mean age at the time of anxiety or depression diagnosis was 65.2 and 64.7 years old, respectively. Pancreatic cancer patients with and without PC consultation had statistically significant differences in the distribution of race, region, Charlson Comorbidity Index scores, concurrent anxiety, or depression compared to those with PC consultation (Table 1). Notably, a higher proportion of patients with anxiety or depression had a PC consultation, compared to those who were not diagnosed with anxiety or depression (36.2% vs. 23.2%, 33.9% vs. 22.8%, respectively, Table 1).

Regardless of mental health diagnosis, most patients in the sample resided in the Midwest and identified as Caucasian (Table 2). A higher proportion of patients with a diagnosis of depression, without anxiety, were over 70 years old, compared to patients with a diagnosis of anxiety alone or both depression and anxiety (Table 2). Notably, for patients consulted by PC who had a diagnosis of anxiety alone, there was a higher prevalence of those who identified as African American, compared to those who were not consulted (10.82% vs. 5.08%, Table 2). This relationship is not observed for those diagnosed with depression and the effect was slightly reversed for those diagnosed with both anxiety and depression (Table 2). When examined by PC consultation within mental health group, the demographic trends remained comparable to those seen with pancreatic cancer patients overall, including the significant differences in comorbidity representation.

There were significant differences in treatment documented by the mental health diagnosis group. For patients with both anxiety and depression, 64.2% documented pharmacotherapy, compared to 52.7% of those with anxiety alone and 39.7% of patients diagnosed with depression alone (*p* < 0.0001, Appendix A. Table A2). As for the utilization of mental health services among the different mental health diagnoses, 35.3% of patients diagnosed with both depression and anxiety documented use of mental health services, compared to 21.8% of those with depression alone and 20.6% of patients with anxiety alone (*p* = 0.0003, Appendix A. Table A2). Regarding the prevalence of treatment among the various mental health diagnoses, stratified by PC consultation, 45.7% of patients diagnosed with both anxiety and depression who were consulted by PC documented the receipt of mental health services, whereas 28.5% of patients with both anxiety and depression but without PC consultation documented mental health services (*p* = 0.0118).

PC had no significant effect on the odds of receiving documented pharmacotherapy, mental health services, or any treatment. Patients diagnosed with anxiety alone or depression alone had lower odds of documented pharmacotherapy (aOR = 0.624 (0.429, 0.907), aOR = 0.365 (0.247, 0.540), respectively), mental health services (aOR = 0.411 (0.266, 0.637), aOR = 0.499 (0.320, 0.779), respectively), and any treatment (aOR = 0.551 (0.371, 0.819), aOR = 0.376; (0.251, 0.564), respectively), compared to patients diagnosed with both anxiety and depression (Table 3). Compared to patients 55–64 years old, patients less than 55 years old had around two times higher odds of documented treatment of any modality and five times higher odds of documented mental health services. A similar trend was seen for patients 55 to 64 years old, who had three times higher odds of documented mental health services than patients 65 to 70 years old. There was no observable association for patients over 70. Patients scoring over 4 on the Charlson Index had higher odds of documented pharmacotherapy (aOR = 1.975 (1.223, 3.188), aOR = 1.583 (1.011, 2.479), respectively, Table 3), and those scoring 4 to 8 had higher odds of any treatment being documented (aOR = 1.866 (1.147, 3.035), Table 3) than those scoring 0 to 3.

## 3. Discussion

Pancreatic cancer ranks as the seventh leading cause of cancer-related deaths worldwide [23]. Due to the advanced stage at the time of diagnosis and the severity of symptoms, palliative care is of utmost importance for pancreatic cancer patients, as it addresses the physical, emotional, and psychosocial needs of patients. Despite known benefits, palliative care utilization remains low among pancreatic cancer patients, consistent with other studies of cancer patients [5,13,24,25]. Our study aims to explore the impact of palliative care consultations on the utilization of mental health services and pharmacotherapy. Our study found significant differences in the distribution of race, region, Charlson Comorbidity Index scores, and concurrent anxiety or depression between pancreatic cancer patients with and without palliative care consultation. Patients consulted for palliative care had a higher proportion of comorbidities and a higher prevalence of anxiety or depression, suggesting that patients with more complex medical and psychological needs are more likely to be referred to palliative care services [26].

Patients with a diagnosis of new-onset depression, without anxiety, were more likely to be over 70 years old, while patients with a diagnosis of new-onset anxiety or both depression and anxiety were younger (Appendix A. Table A5). Moreover, these age-related patterns in mental health diagnoses can also inform further research into the underlying causes and risk factors for these conditions in different age groups. The prevalence of mental health treatment varied depending on the mental health diagnosis. Patients with anxiety alone had a higher prevalence of pharmacotherapy compared to patients with depression alone, whereas patients with anxiety and depression had the highest prevalence of mental health treatment, for both pharmacotherapy and mental health services.

In the multiple regression analysis, we found that patients diagnosed with anxiety alone or depression alone had lower odds of receiving documented pharmacotherapy, mental health services, or any treatment compared to patients diagnosed with both anxiety and depression. These findings suggest that patients with comorbid anxiety and depression may have more severe symptoms and therefore be more likely to receive treatment [27,28]. Studies suggest that depression present in palliative care patients often goes untreated, given that pancreatic cancer patients have a poor prognosis [29]. With a poor prognosis, determining the right dose and appropriate drug for pancreatic cancer patients can pose challenges with limited time. Moreover, we found that younger patients had higher odds of receiving treatment, with patients less than 55 years old having around two times higher odds of documented treatment compared to patients 65 to 70 years old, consistent with existing literature surrounding mental healthcare delivery to younger and older patients [30].

The absence of a significant association between palliative care and the likelihood of any documented treatment may be attributed to several factors. It is possible that palliative care consultations primarily addressed physical symptoms rather than focusing on mental health symptoms. A study by Hatano et al. in 2018 found that pain management was the primary reason for PC deferral, and depression was a secondary, yet less common reason. Furthermore, treatment was often withheld because of very late referrals [31]. Additionally, patients might not have prioritized mental health as a primary aspect of their care during these PC consultations.

Despite these important findings, there are several limitations to be noted. We could not determine the staging of pancreatic cancer given the nature of the administrative data source utilized in this study. Moreover, the specific focus on anxiety and depression, while important, might exclude other pertinent mental health conditions, such as acute stress reactions following cancer diagnosis, post-traumatic stress disorder, or substance use disorders, therefore reducing the comprehensive assessment of mental health in this context. Lastly, the actual receipt of pharmacotherapy and mental health is challenging to ascertain in the EHR database, leaving some possibility of misclassification.

Regardless of these limitations, this study has several notable strengths. First, we utilized a comprehensive approach to ICD-9/10 codes, which have been demonstrated in prior work to be highly sensitive [19]. The data source used for the study has both commercially and Medicare-insured patients from diverse regions across the United States and depicts a high degree of representativeness for our study, thereby rendering our findings generalizable to the broader population. These findings also provide invaluable insights into the demographics and mental health attributes of this patient group, offering healthcare professionals a deeper understanding to develop tailored interventions for pancreatic cancer patients.

The overall comprehensive approach, exploring sociodemographic factors, like race, age, gender, region, comorbidity index scores, and mental health diagnoses, yields a depiction of demographics and mental health profiles of pancreatic cancer patients. Notably, this study underscores the importance of addressing both anxiety and depression together within the scope of pancreatic cancer treatment, emphasizing the fundamental necessity for a more comprehensive and integrated approach to patient-centered care.

## 4. Conclusions

Our study highlights the need for further investigation of palliative care in relation to mental health treatments and services utilization for pancreatic cancer patients. Furthermore, our study findings can serve as a valuable resource for healthcare providers, enabling them to make informed decisions regarding the implementation of palliative care.

## Figures and Tables

**Table 1:** Sample Characteristics Stratified by Receipt of Palliative Care Consultation.

	Newly Diagnosed Pancreatic Cancer with Palliative Care Consults	Newly Diagnosed Pancreatic Cancer without Palliative Care Consults	
	*n* = 1022	*n* = 3007	*p*-Value
**Gender (%)**			0.7651
Female	50.39	49.85	
Male	49.61	50.15	
**Race (%)**			0.0153
Caucasian	80.53	80.51	
African	11.45	9.15	
Other	8.02	10.34	
**Age at Cancer Diagnosis (years) (%)**			0.5168
<55	13.41	14.4	
55–64	23.19	21.98	
56–70	15.56	17.03	
>70	47.85	46.59	
**Region (%)**			0.0344
Midwest	51.57	46.49	
Northeast	14.97	14.83	
South	22.02	24.81	
West	8.81	11.07	
Other/Unknown	2.64	2.79	
**Charlson Comorbidity Index (%)**			<0.0001
0–3	7.44	37.35	
4–8	22.41	28.2	
>8	70.16	34.45	
**Depression (%)**			<0.0001
Yes	33.95	22.85	
**Anxiety (%)**			<0.0001
Yes	36.2	23.21	

**Table 2:** Sample Characteristics, Grouped by Mental Health Diagnoses and Stratified by Receipt of Palliative Care Consultation.

	New Onset Depression			New Onset Anxiety			Both Anxiety and Depression		
	With PC	Without PC	*p*-Value	With PC	Without PC	*p*-Value	With PC	Without PC	*p*-Value
	*n* = 88	*n* = 169		*n* = 177	*n* = 119		*n* = 81	*n* = 123	
**Gender (%)**			0.3141			0.0585			0.116
Female	54.55	47.93		48.74	59.89		45.68	56.91	
Male	45.45	52.07		51.26	40.11		54.32	43.09	
**Race (%)**			0.0838			0.1714			0.4823
Caucasian	87.5	78.7		84.87	90.4		91.36	89.43	
African	10.23	11.83		10.82	5.08		3.7	7.32	
Other	2.27	9.47		4.2	4.52		4.94	3.25	
**Age at Cancer Diagnosis (years) (%)**			0.3296			0.9823			0.481
<55	17.05	13.02		15.97	15.82		22.22	26.02	
55–64	30.68	26.04		31.93	30.51		24.69	23.58	
65–70	9.09	16.57		15.97	15.25		19.75	12.2	
>70	43.18	44.38		36.13	38.42		33.33	38.21	
**Region (%)**			0.4073			0.1938			0.238
Midwest	51.14	52.07		50.42	50.28		39.51	53.66	
Northeast	17.05	9.47		20.17	13.56		17.28	9.76	
South	21.59	24.26		21.01	23.16		29.63	27.64	
West	7.95	9.47		5.04	11.3		9.88	7.32	
Other/Unknown	2.27	4.73		3.36	1.69		3.7	1.63	
**Charlson Comorbidity Index (%)**			<0.0001			0.0003			<0.0001
0–3	6.82	20.71		5.88	21.47		1.23	23.58	
4–8	20.45	34.32		26.89	30.51		14.81	30.89	
>8	72.73	44.97		67.23	48.02		83.95	45.53	

**Table 3:** Results of Multiple Logistic Regression for Mental Health Service Use, Pharmacotherapy, and Any Treatment.

	Any Treatment	Pharmacotherapy	Mental Health Services
	Odds Ratio (95% CI)	Odds Ratio (95% CI)	Odds Ratio (95% CI)
**Palliative Care**	0.806 (0.581, 1.117)	0.766 (0.556, 1.055)	1.410 (0.963, 2.064)
**Mental Health Diagnosis**			
New—Onset Anxiety	0.551 (0.371, 0.819)	0.624 (0.429, 0.907)	0.411 (0.266, 0.637)
New—Onset Depression	0.376 (0.251, 0.564)	0.365 (0.247, 0.540)	0.499 (0.320, 0.779)
Both (ref)	—	—	—
**Age at Cancer Diagnosis (years)**			
<55	1.832 (1.058, 3.171)	1.449 (0.853, 2.462)	5.18 (2.557, 10.492)
55–64	1.360 (0.837, 2.209)	1.024 (0.635, 1.650)	3.015 (1.528, 5.948)
65–70 (ref)	—	—	—
>70	1.021 (0.650, 1.606)	0.914 (0.583, 1.431)	1.883 (0.961, 3.688)
**Gender**			
Female (ref)	—	—	—
Male	1.054 (0.777, 1.430)	1.133 (0.841, 1.527)	0.960 (0.670, 1.374)
**Race**			
Caucasian (ref)	—	—	—
African	0.881 (0.508, 1.531)	0.991 (0.574, 1.711)	1.385 (0.736, 2.609)
Other	0.724 (0.364, 1.438)	0.788 (0.398, 1.561)	0.590 (0.238, 1.462)
**Region**			
Northeast (ref)	—	—	—
Midwest	0.680 (0.420, 1.101)	1.044 (0.667, 1.635)	0.569 (0.353, 0.915)
South	0.458 (0.269, 0.778)	0.808 (0.490, 1.333)	0.158 (0.083, 0.300)
West	0.351 (0.181, 0.679)	0.598 (0.314, 1.139)	0.311 (0.140, 0.689)
Other/Unknown	0.538 (0.205, 1.412)	0.808 (0.312, 2.093)	0.644 (0.232, 1.788)
**Charlson Comorbidity Index**			
0–3 (ref)	—	—	—
4–8	1.866 (1.147, 3.035)	1.975 (1.223, 3.188)	1.397 (0.777, 2.509)
>8	1.423 (0.906, 2.234)	1.583 (1.011, 2.479)	1.351 (0.776, 2.350)

## Data Availability

Data is available through Optum.

## Appendix A

**Table A1:** ICD-9 and ICD-10 Codes for Diagnoses of Depression and Anxiety.

ICD-9	Depression Diagnosis
296.2	Major depressive affective disorder, single episode, unspecified
296.21	Major depressive affective disorder, single episode, mild
296.22	Major depressive affective disorder, single episode, moderate
296.23	Major depressive affective disorder, single episode, severe w/o psychotic features
296.24	Major depressive affective disorder, single episode, severe w/ psychotic features
296.25	Major depressive affective disorder, single episode, partial remission
296.26	Major depressive affective disorder, single episode, full remission
311	Depression, unspecified
296.3	Major depressive affective disorder, recurrent, unspecified
296.31	Major depressive affective disorder, recurrent, mild
296.32	Major depressive affective disorder, recurrent, moderate
296.33	Major depressive affective disorder, recurrent, severe w/o psychotic features
296.34	Major depressive affective disorder, recurrent, sever w/ psychotic features
296.35	Major depressive affective disorder, recurrent, in remission (unspecified)
296.35	Major depressive affective disorder, recurrent, in remission (partial)
296.36	Major depressive affective disorder, recurrent, in remission (full)
**ICD-10**	**Depression Diagnosis**
F32.0	MDD, single episode, mild
F32.1	MDD, single episode, moderate
F32.2	MDD, single episode, severe w/o psychotic features
F32.3	MDD, single episode, severe w/ psychotic features
F32.4	MDD, single episode, partial remission
F32.5	MDD, single episode, full remission
F32.8	Other depressive episodes
F32.81	Premenstrual dysphoric disorder
F32.89	Other specified depressive episodes
F32.9	MDD, single episode, unspecified
F32.A	Depression, unspecified
F33.0	MDD, recurrent, mild
F33.1	MDD, recurrent, moderate
F33.2	MDD, recurrent, severe w/o psychotic features
F33.3	MDD, recurrent, sever w/ psychotic features
F33.4	MDD, recurrent, in remission
F33.40	MDD, recurrent, in remission (unspecified)
F33.41	MDD, recurrent, in remission (partial)
F33.42	MDD, recurrent, in remission (full)
F33.8	Other recurrent depressive disorders
F33.9	MDD, recurrent, unspecified
**ICD-9**	**Anxiety Diagnosis**
300.01	Panic Disorder
300.02	Generalized Anxiety Disorder
300	Anxiety State, unspecified
300.09	Other anxiety states
**ICD-10**	**Anxiety Diagnosis**
F41.0	Panic Disorder
F41.1	Generalized Anxiety Disorder
F41.3	Other mixed anxiety disorders
F41.8	Other specified anxiety disorders
F41.9	Anxiety disorder, unspecified
**ICD-10**	**Other Mental Health Disorder due to known physiological condition**
F06.3	Mood Disorder due to known physiological condition
F06.30	Mood Disorder due to known physiological condition (unspecified)
F06.31	Mood Disorder due to known physiological condition (w/ depressive features)
F06.32	Mood Disorder due to known physiological condition (with major depressive like episode)
F06.33	Mood Disorder due to known physiological condition (with manic features)
F06.34	Mood Disorder due to known physiological condition (with mixed features)
F06.4	Anxiety disorder due to known physiological condition
**ICD-9**	**Unspecified**
296.9	Unspecified mood disorder
300.9	Unspecified mental disorder
**ICD-10**	**Unspecified**
F39	Unspecified mood disorder
F99	Unspecified mental disorder

**Table A2:** CPT Codes Used to Measure Utilization of Mental Health Services.

CPT	Procedure Heading
	**Psychiatric Diagnostic Procedures**
90791	Psychiatric diagnostic evaluation
90792	Psychiatric diagnostic evaluation with medical services
90801 *	Psychiatric diagnostic interview examination
	**Psychotherapy**
90804 *	Outpatient psychotherapy, 20–30 min
90816 *	Inpatient psychotherapy, 20–30 min
90804 *	Outpatient psychotherapy, 45–50 min
90818 *	Inpatient psychotherapy, 54–50 min
90808 *	Outpatient psychotherapy, 75–80 min
90821 *	Inpatient psychotherapy, 75–80 min
90832	Psychotherapy, 30 min with patient
90833	Psychotherapy, 30 min with patient w/E and M service
90834	Psychotherapy, 45 min with patient
90836	Psychotherapy, 45 min with patient w/E and M service
90837	Psychotherapy, 60 min with patient
90838	Psychotherapy, 60 min with patient w/E and M service
90845 *	Psychoanalysis
90846 *	Family psychotherapy w/o patient present
90847 *	Family psychotherapy, conjoint w/ patient present
90849 *	Multiple family group psychotherapy
90853 *	Group psychotherapy (other than of a multiple family group)
	**Psychotherapy for crisis**
90839	Psychotherapy for crisis; first 60 min
90840	Psychotherapy for crisis; each additional 30 min
	**Other Psychotherapy**
90845	Psychoanalysis
90846	Family psychotherapy w/o patient, 50 min
90847	Family psychotherapy w/ patient, 50 min
90849	Multiple-family group psychotherapy
90853	Group psychotherapy (other than of a multiple group family)
	**Other Psychiatric Services or Procedures**
90863	Pharmacologic management, including prescription review of medication, when performed with psychotherapy services
90865	Narcosynthesis for psychiatric diagnostic and therapeutic purposes
90867	Therapeutic repetitive transcranial magnetic stimulation treatment; initial, including cortical mapping, motor threshold determination, delivery and management
90868/9	TMS, subsequent delivery and management, per session
90870	ECT
90875	Individual psychophysiological therapy incorporating biofeedback training by any modality, with psychotherapy; 30 min
90875	Additional 45 min
90880	Hypnotherapy
90882	Environmental intervention for medical management purposes on a psychiatric patient’s behalf with agencies, employers, or institutions
90885	Psychiatric evaluation of hospital records, other psychiatric reports, psychometric and/or projective tests, and other accumulated data for medical diagnostic purposes
90887	Interpretation or explanation of results of psychiatric, other medical examinations and procedures, or other accumulated data to family or other responsible persons, or advising them how to assist patient
90889	Preparation of report of patient’s psychiatric status, history, treatment, or progress for other individuals, agencies, or insurance carriers
90899	Unlisted psychiatric service or procedure
	**Psychiatric Collaborative Care Management Services**
99492	Initial psychiatric collaborative care management
99493	Subsequent psychiatric collaborative care management
99494	Initial of subsequent psychiatric collaborative care management
	**Codes for Interactive Services**
90802 *	Interactive psychiatric diagnostic evaluation
90857 *	Interactive group psychotherapy
	**Pharmacologic Management add-on code**
90862 *	Pharmacologic management, including prescription, use and review of medication with no more than minimal medical psychotherapy

* Code discontinued.

**Table A3:** Pharmacotherapy for Anxiety.

Drug Class	Generic Name
SSRI	Citalopram
	Escitalopram
	Fluoxetine
	Fluvoxamine
	Paroxetine
	Sertraline
	Vilazodone
	Vortioxetine
	Nefazodone
	Trazodone
SNRI	Desvenlafaxine
	Duloxetine
	Levomilnacipran
	Milnacipran
	Venlafaxine
Benzodiazepine	Alprazolam
	Alprazolam
	Bromazepam
	Chlordiazepoxide
	Clonazepam
	Clorazepate
	Diazepam
	Lorazepam
	Oxazepam
	Prazepam
Other	Buspirone
	Gabapentin
	Pregabalin
	Mirtazapine
	Quetiapine
	Hydroxyzine
	Imipramine

Abbreviations: selective-serotonin reuptake inhibitor (SSRI), serotonin and norepinephrine reuptake inhibitor (SNRI).

**Table A4:** Pharmacotherapy for Depression.

Drug Class	Generic Name
SSRI	Citalopram
	Escitalopram
	Fluoxetine
	Fluvoxamine
	Paroxetine
	Sertraline
	Vilazodone
	Vortioxetine
	Nefazodone
	Trazodone
SNRI	Desvenlafaxine
	Duloxetine
	Levomilnacipran
	Milnacipran
	Venlafaxine
TCA	Amitriptyline
	Amoxapine
	Clomipramine
	Desipramine
	Doxepin
	Imipramine
	Maprotiline
	Nortriptyline
	Protriptyline
	Trimipramine
MAO-i	Isocarboxazid
	Phenelzine
	Selegiline
	Tranylcypromine
Other	Bupropion
	Esketamine
	Mirtazapine

Abbreviations: selective-serotonin reuptake inhibitor (SSRI), serotonin and norepinephrine reuptake inhibitor (SNRI), tricyclic antidepressant (TCA), monoamine-oxidase inhibitor (MAO-i).

**Table A5:** Sample Characteristics Stratified by Mental Health Diagnosis.

	New-Onset Anxiety	New-Onset Depression	Both Anxiety and Depression	
	*n* = 296	*n* = 257	*n* = 204	*p*-value
**Gender (%)**				0.4686
Female	55.41	50.19	52.45	
Male	44.59	49.81	47.55	
**Race (%)**				0.0848
Caucasian	88.18	81.71	90.2	
African	7.43	11.28	5.88	
Other	4.39	7	3.92	
**Age at Cancer Diagnosis (years) (%)**				0.0614
<55	15.88	14.4	24.51	
55–64	31.08	27.63	24.02	
65–70	15.54	14.01	15.2	
>70	37.5	43.97	36.27	
**Region (%)**				0.6863
Midwest	50.34	51.75	48.04	
Northeast	16.22	12.06	12.75	
South	22.3	23.35	28.43	
West	8.78	8.95	8.33	
Other/Unknown	2.36	3.89	2.45	
**Charlson Comorbidity Index (%)**				0.6946
0–3	15.2	15.95	14.71	
4–8	29.05	29.57	24.51	
>8	55.74	54.47	60.78	
**Treatment (%)**				
Pharmacotherapy	52.7	39.69	64.22	<0.0001
Mental Health Services	20.61	21.79	35.29	0.0003
Any Treatment	59.46	49.42	72.06	<0.0001
